# Short-Term Sleep Deprivation Stimulates Hippocampal Neurogenesis in Rats Following Global Cerebral Ischemia/Reperfusion

**DOI:** 10.1371/journal.pone.0125877

**Published:** 2015-06-03

**Authors:** Oumei Cheng, Rong Li, Lei Zhao, Lijuan Yu, Bin Yang, Jia Wang, Beibei Chen, Junqing Yang

**Affiliations:** 1 Department of Neurology, The First Affiliated Hospital, Chongqing Medical University, Chongqing 400016, China; 2 Department of Pharmacology, Chongqing Medical University, the Key Laboratory of Biochemistry and Molecular Pharmacology, Chongqing 400016, China; Georgia Regents University, Medical College of Georgia, UNITED STATES

## Abstract

**Background:**

Sleep deprivation (SD) plays a complex role in central nervous system (CNS) diseases. Recent studies indicate that short-term SD can affect the extent of ischemic damage. The aim of this study was to investigate whether short-term SD could stimulate hippocampal neurogenesis in a rat model of global cerebral ischemia/reperfusion (GCIR).

**Methods:**

One hundred Sprague-Dawley rats were randomly divided into Sham, GCIR and short-term SD groups based on different durations of SD; the short-term SD group was randomly divided into three subgroups: the GCIR+6hSD*3d-treated, GCIR+12hSD-treated and GCIR+12hSD*3d-treated groups. The GCIR rat model was induced via the bilateral occlusion of the common carotid arteries and hemorrhagic hypotension. The rats were sleep-deprived starting at 48 h following GCIR. A Morris water maze test was used to assess learning and memory ability; cell proliferation and differentiation were analyzed via 5-bromodeoxyuridine (BrdU) and neuron-specific enolase (NSE), respectively, at 14 and 28 d; the expression of hippocampal BDNF was measured after 7 d.

**Results:**

The different durations of short-term SD designed in our experiment exhibited improvement in cognitive function as well as increased hippocampal BDNF expression. Additionally, the short-term SD groups also showed an increased number of BrdU- and BrdU/NSE-positive cells compared with the GCIR group. Of the three short-term SD groups, the GCIR+12hSD*3d-treated group experienced the most substantial beneficial effects.

**Conclusions:**

Short-term SD, especially the GCIR+12hSD*3d-treated method, stimulates neurogenesis in the hippocampal dentate gyrus (DG) of rats that undergo GCIR, and BDNF may be an underlying mechanism in this process.

## Introduction

Global cerebral ischemia/reperfusion (GCIR), a syndrome characterized by the rapid interruption of cerebral blood flow, occurs in patients who suffer from cardiac arrest, shock or complex cardiac surgery and is usually accompanied by a broad range of neuronal death in the brain[1–3]. Many of these patients suffer from various degrees of memory loss and learning dysfunction, suggesting an impairment in the hippocampus, which is the primary region of the brain that controls the formation of memories and learned behaviors[4, 5]. Currently, counteracting ischemia-induced cognitive impairment is challenging, and effective strategies that can attenuate the negative effects of ischemia are insufficient. The increased neurogenesis, especially in the hippocampus of adults after GCIR, has been reported to be a compensatory adaptive response to brain injury that could counteract the negative effects of cell death and cognitive dysfunction[6].

Sleep is necessary for health and survival. After decades of research into the function the sleep-wake cycle, a large body of evidence strongly indicates that sleep deprivation (SD) has complex detrimental consequences on rodents and humans, though the mechanisms underlying these negative effects remain largely unclear[7–10]. However, growing evidence also suggests that short-term sleep deprivation (SD) with a duration of less than 48 h produces positive effects. For example, previous studies suggest that 6–12 h of short-term SD prior to cerebral ischemia produces neuroprotective effects by attenuating inflammatory responses and glial reactions in the rat hippocampus[11–13]. Moreover, a recent study showed that 24 h of short-term SD immediately following traumatic brain injury (TBI) reduces morphological damage and enhances recovery in rats[14]. Hence, we suggest that the effect of sleep deprivation may largely depend on the time window and duration of SD. More specially, emerging studies have also reported that 12 h of short-term SD can promote neurogenesis in the hippocampus of normal rats[15, 16]. Based on these findings, we hypothesized that short-term SD may improve cognitive function in an ischemic model through neurogenesis-induced neuronal regeneration. Similarly, the ideal durations and time window of SD is significant.

Brain-derived neurotrophic factor (BDNF) has recently been shown to be a homeostatic regulator of sleep [17–19]. BDNF is also known to increase neurogenesis, neurite sprouting and other processes related to the general enhancement of hippocampal function in normal and ischemic rodents[20]. Some reports have demonstrated that short-term SD increases BDNF expression in the hippocampus in normal rats[15]. We speculate that the BDNF pathway mediates the neurogenesis induced by short-term SD, thereby ameliorating cognitive function in global ischemia.

In light of this, the present study aimed to determine whether different durations of short-term SD could stimulate hippocampal neurogenesis and ameliorate the impaired cognitive functions induced via GCIR in rats in an appropriate time window and whether this self-repair is related to the hippocampal BDNF pathway.

## Materials and Methods

### Animal groups

One hundred male Sprague—Dawley rats (weighing 230±20 g), were purchased from the Experimental Animal Center of Chongqing Medical University. They were housed in groups of five in polycarbonate cages under a 12:12 h light:dark cycle (lights on from 9:00 to 21:00) with unlimited access to food and water at an ambient temperature of 21±1°C and a relative humidity of 40–50%. All experiments were approved by the Chongqing Medical University Institutional Lab Animal Care and Use Committee and were in accordance with the National Institutes of Health guidelines.

The rats were maintained in the institutional animal facilities for at least 2 weeks and then randomly assigned to 3 groups: the Sham group (n = 20), the GCIR group (n = 20), and the short-term SD group. Based on the different durations of SD, the short-term SD group was randomly divided into three subgroups: the GCIR+6hSD*3d-treated group (n = 20) rats were subjected to GCIR followed by sleep deprivation for 6 h/d continuously for 3 d; the GCIR+12hSD-treated group (n = 20), rats were subjected to GCIR followed by sleep deprivation for 12 h; and the GCIR+12hSD*3d-treated group (n = 20) rats were subjected to GCIR followed by sleep deprivation treatment for 12 h/d continuously for 3 d. The rats were sleep-deprived starting at 48 h following GCIR.

### Global ischemia reperfusion model establishment

Global cerebral ischemia/reperfusion (GCIR) modeling was performed in a manner similar to a previous method with slight modifications [21, 22]. Briefly, GCIR was induced via ligation of the bilateral common carotid artery combined with hemorrhagic hypotension in rats. The rats underwent fasting for 12 hours prior to surgery. After anesthesia was induced (3.5% chloral hydrate, 1 ml/100 g, i.p.), the bilateral common carotid arteries were slightly isolated, and the right jugular vein was cannulated with tubing connected to a heparinized syringe. Blood (2.5 ml/100 g) was slowly withdrawn from the right jugular vein until the volume reached standard requirements, at which time the bilateral common carotid arteries were temporarily occluded using artery clamps for 20 min; the extracted blood was then slowly reinfused, and the catheters were withdrawn. The rats in the sham group were subjected to the same operation described above, with the exception of the bilateral carotid artery occlusion and hemospasia from the right jugular vein. We discussed the success and exclusion criteria of the GCIR model in our previous report [22].

### Sleep deprivation procedures

Sleep deprivation was conducted 48 h following GCIR using the modified multiple platform method, as described previously, which does not involve immobility stress or forced activity, resulting in less interference from other factors[23, 24]. Sleep deprivation was initiated at 09:00AM during the rest circadian phase of the rats (light phase: 09:00AM-9:00PM). Briefly, groups of 8 rats were placed in water tanks (75×34×17 cm) containing 8 small circular platforms (6.5 cm in diameter). The surfaces of all platforms were 1 cm above the water level. The rats fall into the water if they lose muscle tonus, forcing them to climb back onto the platform, thus being awakened. Additionally, the rats in the sham and GCIR groups were placed in an identical apparatus that was equipped with a larger platform (18 cm in diameter) to permit sleep, serving as a control for the small platform to filter out the effects of nonspecific stressors. All of the rats were placed on the platform for 10 min twice per day beginning one week prior to the experiment to adapt to the homemade device. Moreover, food and water were provided ad libitum throughout the study, and the water in the tanks was changed daily.

### BrdU Incorporation

BrdU (B5002, Sigma Aldrich, Munich, Germany) was dissolved at a concentration of 10 mg/ml in fresh isotonic sterile saline prepared just prior to use and the solution was injected in a volume of 5 ml/kg/d of the body weight. And BrdU was injected four times with a 2 h interval in the 13th day and 27th day after GCIR. Rats were sacrificed 24h after the last BrdU injection.

### Morris Water Maze Test

The Morris water maze (MWM) procedure was employed to assess spatial learning and memory function of the rats 7 days after the beginning of SD procedure and included 5 days of spatial acquisition and 1 day of a probe trial[25]. The MWM (Institute of Materia Medica, Chinese Academy of Medica Sciences, Beijing, China) was equipped with a diameter of 150 cm, height of 50 cm, water depth of 40 cm, and temperature of 24±1°C. A platform (10 cm in diameter) was submerged 1 cm below the surface of the water and placed in the middle of the same quadrant throughout the training phase. During the learning process (1–5 d), the rats (n = 6 per group) were subjected to four consecutive trials per day with intervals of 5 min. In each trial, an individual rat was placed into the pool and permitted to search for the submerged platform for 90 s. If a rat failed to locate the platform within 90 s, it would be gently guided to the platform, and the escape latency was recorded as 90 s. The mean escape latency of 4 trials was noted as the daily result of learning ability for the animal. On the 6th day of the test, each rat was placed into the pool after the platform had been previously removed and then allowed to explore the pool for 90 s. The frequency with which each rat passed the hidden platform and the resident time that each rat spent in the target quadrant were noted as the result of the spatial memory function.

### Preparation of paraffin-embedded tissue

Four rats were selected in each group for histopathological observation respectively at the 14 d and 28 d timepoints after GCIR. Rats were anesthetized with 3.5% chloral hydrate (1 ml/100 g, i.p.) and rapidly perfused with ice-cold saline (approximately 200 ml. Then, rats were perfused with 250 ml of 4% paraformaldehyde until the liver became pale and the limbs and neck became straight and stiff. Finally, paraffin sections of coronal slices in the hippocampal dentate gyrus (DG) were cut with a slice thickness of 4μm.

### Preparation of fresh tissue

On the 7th day after GCIR, the rats (n = 6) were decapitated, and their hippocampi were rapidly removed on ice, quickly frozen in liquid nitrogen and stored at -80°C in a refrigerator until ELISA.

### Immunohistochemistry and immunofluorescence analysis

Briefly, brains embedded in paraffin were used to examine BrdU incorporation 14 d after GCIR to assess the proliferation of newly generated cells. The sections were immersed in 3% H2O2 for 30 min at 37°C to block endogenous peroxidase activity. After being washed in PBS, the sections were blocked with 5% BSA for 30 min, incubated with a primary mouse monoclonal antibody against BrdU (1:50,B2631,Sigma)overnight at 4°C, and then incubated with biotinylated goat anti-mouse IgG(1:100,Zhongshan Inc) for 60 min. Immunoreactivity was detected with 0.05% diaminobenzidine (DAB) containing 0.03% H2O2 for 5 min.

To further determine the cell lineage of the proliferating cells, we examined the expression of BrdU and the neuronal marker neuron-specific enolase (NSE) 28 d after GCIR [26, 27]. After pretreatment with 2 N HCl, the sections prepared as described above were incubated overnight at 4°C in anti-BrdU (1:50, B2631, Sigma) and rabbit anti-NSE antibodies (1:300; Cell Signaling Technology, Danvers, MA) and then incubated for 1 h at room temperature in Alexa Fluor 488 goat anti-mouse IgG(1:500, Invitrogen, Carlsbad, CA) and Alexa Fluor 594 goat anti-rabbit IgG (1:500, Invitrogen, Carlsbad, CA) for 1 h at room temperature. The sections were washed with PBS-T 3×10 min followed by 2× 5 min washes with PBS and 2×1 min washes with water; they were then mounted with water-based mounting medium containing anti-fading agents (Biomeda, Fischer Scientific, Pittsburgh, PA).

### Image Analysis

The sections were examined by light microscopy (BX51, Olympus, Tokyo, Japan). Cells were counted, in a blinded manner, within defined regions of interest in the inner edge of the granule cell layer (GCL) of the DG. Cell counting in hippocampal DG was performed in the ipsilateral DG using a series of coronal sections between 10.0 mm and 10.6 mm from the front of the brain. In total, six 4-μm coronal sections were examined per animal (n = 4 per group), spaced 100μm apart, under high-power (40×objective) using Image-Pro Plus 6.0 for Windows (Media Cybernetics, MD, USA). In each section, measurements were made in ten regions of interest. The planar area enclosed by each region was 50μm×50μm. The average ratio of BrdU-positive cells in the proportion of the total number of cells and the average ratio of BrdU/NSE-positive cells in the proportion of BrdU-positive cells in the DG per section were respectively calculated.

### BDNF ELISA

The hippocampal tissue was placed in Eppendorf tubes and immersed in isopentane cooled by dry ice. This process did not take longer than 3 minutes. These samples were homogenized with a Dounce homogenizer (Kontes-7 ml, Vineland, NJ, USA) in ice-cold homogenate buffer solution. The ELISA procedure was performed according to the manufacturer’s instructions (BOSTER, China) using a microplate reader (Bio-Rad, Richmond, CA, USA).

### Statistical analysis

The results were expressed as the means ± standard deviation of the means. Two-factor multi-level analysis of repeated measures was used for the comparison of the water maze escape latencies. Statistical comparisons of differences between groups for different interventions were performed using one-way ANOVA. All statistical analyses were performed with the Statistical Package for the Social Sciences (SPSS 10.0) software, and p<0.05 was considered statistically significant.

## Results

### Effect of short-term sleep deprivation on spatial learning and memory ability in rats with GCIR

**Fig 1:**
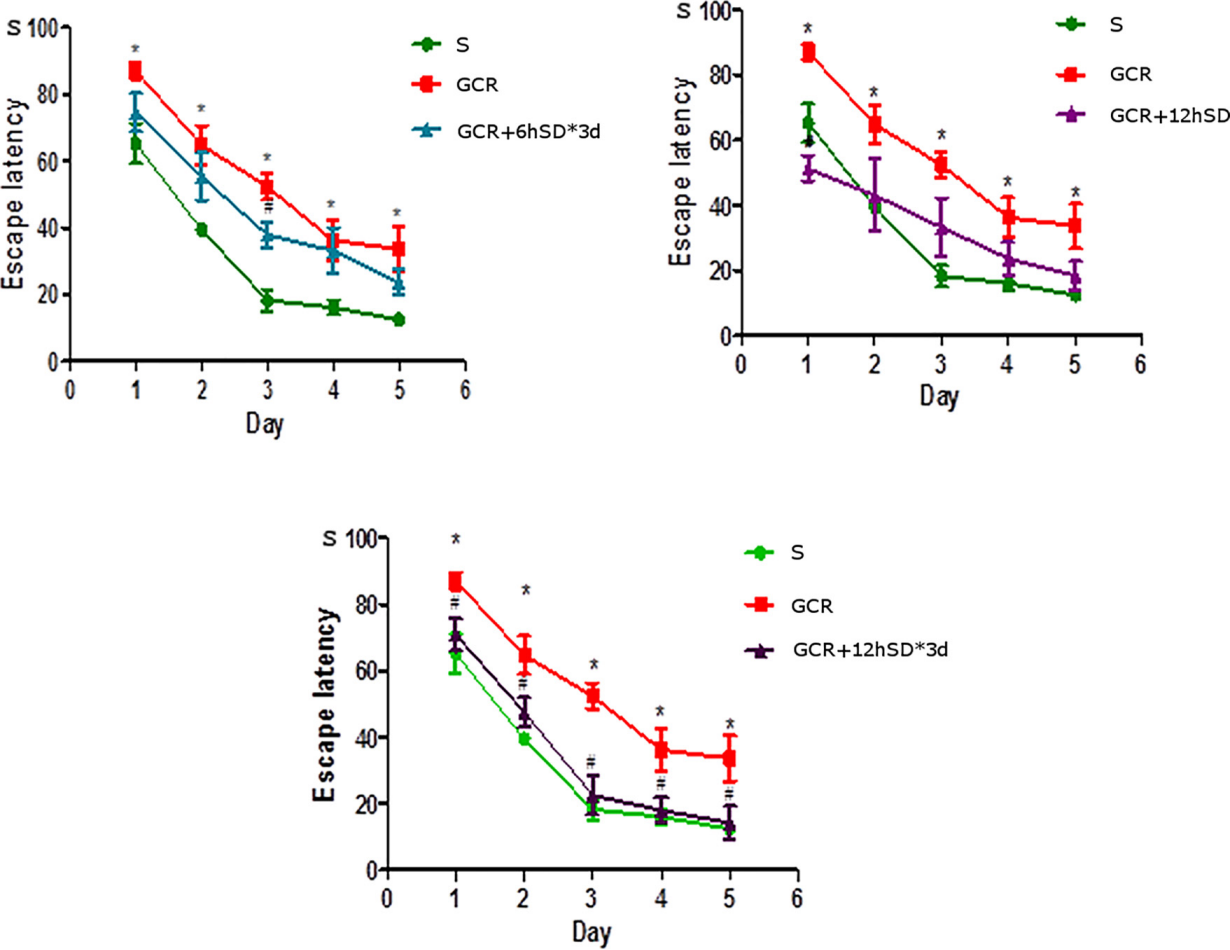
Effect of short-term SD on escape latency of rat after GCIR ($\bar{\text{x}}\pm \text{S}$, n = 6). The rats in the GCIR group spent longer in their escape latencies to find the platform than the rats in the S group during the five training days. The rats in the GCIR+12hSD*3d group exhibited a significantly shorter escape latency compared with the GCIR group during the five training days. * P<0.05 compared with the S group; # P<0.05 compared with the GCIR group.

The Morris water maze results showed that rats in all the five groups exhibited a rapid reduction in their escape latencies to find the platform over the five training days (Fig 1). Compared with the S group, the rats subjected to GCIR showed a prolonged escape latency, which implies that global ischemia significantly impaired spatial learning ability, and this impairment occurred from training day 1 onward (p<0.05). Surprisingly, there was no apparent difference between the escape latency of rats in the GCIR+12hSD group and the GCIR group. And the rats in the GCIR+6hSD*3d group just spent less escape latency than the rats in the GCIR group on day 3 (p<0.05). However, the escape latency in the GCIR+12hSD*3d group was significantly shorter than which in the GCIR group, and the decrease trend substantially occurred in training day 1 onward (p<0.05), which indicates that the 12hSD*3d-treated method can improve the spatial learning ability of rats with global cerebral ischemia/reperfusion-induced injury.

**Fig 2:**
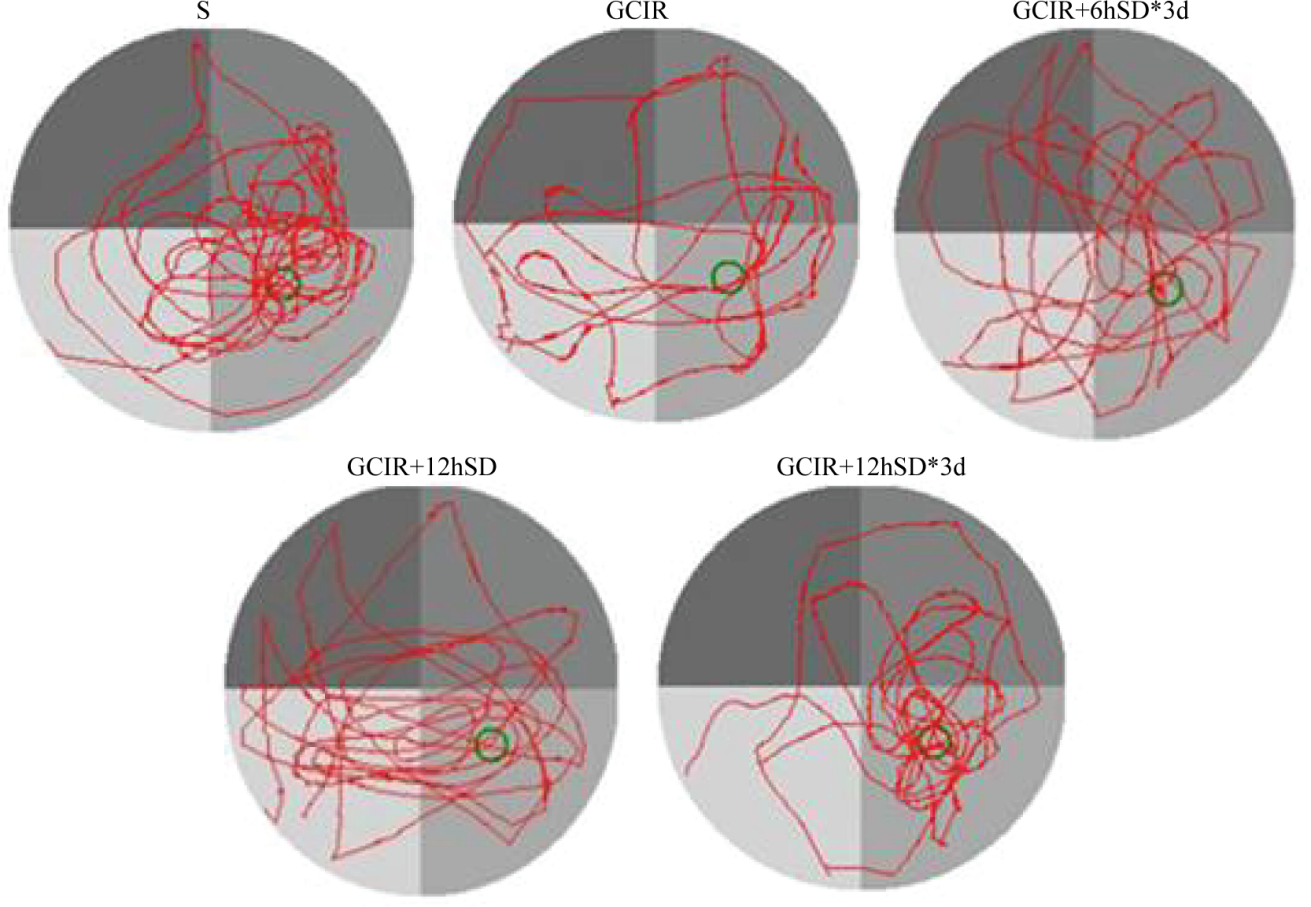
Typical trajectories of the orientation test on day 6 of the water maze test. The green circle in the trace images shows the previous platform location.

During the probe test, the platform was removed, then the resident time and frequency with which the rats crossed the target quadrant was recorded (Table 1 and Fig 2). Relative to the S group, the rats with GCIR showed less crossing times and resident time in the test (p<0.01). However, the rats in all short-term SD groups (including GCIR+6hSD*3d, GCIR+12hSD, and GCIR+12hSD*3d) spent significant longer and made substantial more frequent cross-platform movements in the original platform quadrant than the rats in the GCIR group (respectively p<0.05, p<0.01, p<0.01). This indicates that the rats in the S and all short-term SD groups exhibited better spatial memory regarding the original platform location.

**Table 1:** Effect of short-term SD on residence time and target crossing of rats after GCIR ($\bar{\text{x}}\pm \text{S}$, n = 6).

Groups	S	GCIR	GCIR+SD6h*3d	GCIR +SD12h	GCIR+SD12h*3d
Residence time	85.34±11.28	47.26±12.41**	59.55±12.37** #	64.30±16.83** ##	76.25±17.62* ##
Target crossing	9.53±2.46	3.42±0.72**	5.13±1.43** #	6.38±1.81** ##	8.14±1.20* ##

* P<0.05

** P<0.01compared with the S group

^#^ P<0.05

^##^ P<0.01 compared with the GCIR group

### Effect of short-term sleep deprivation on cell proliferation in the dentate gyrus (DG) of the hippocampus in rats 14 d after GCIR

**Fig 3:**
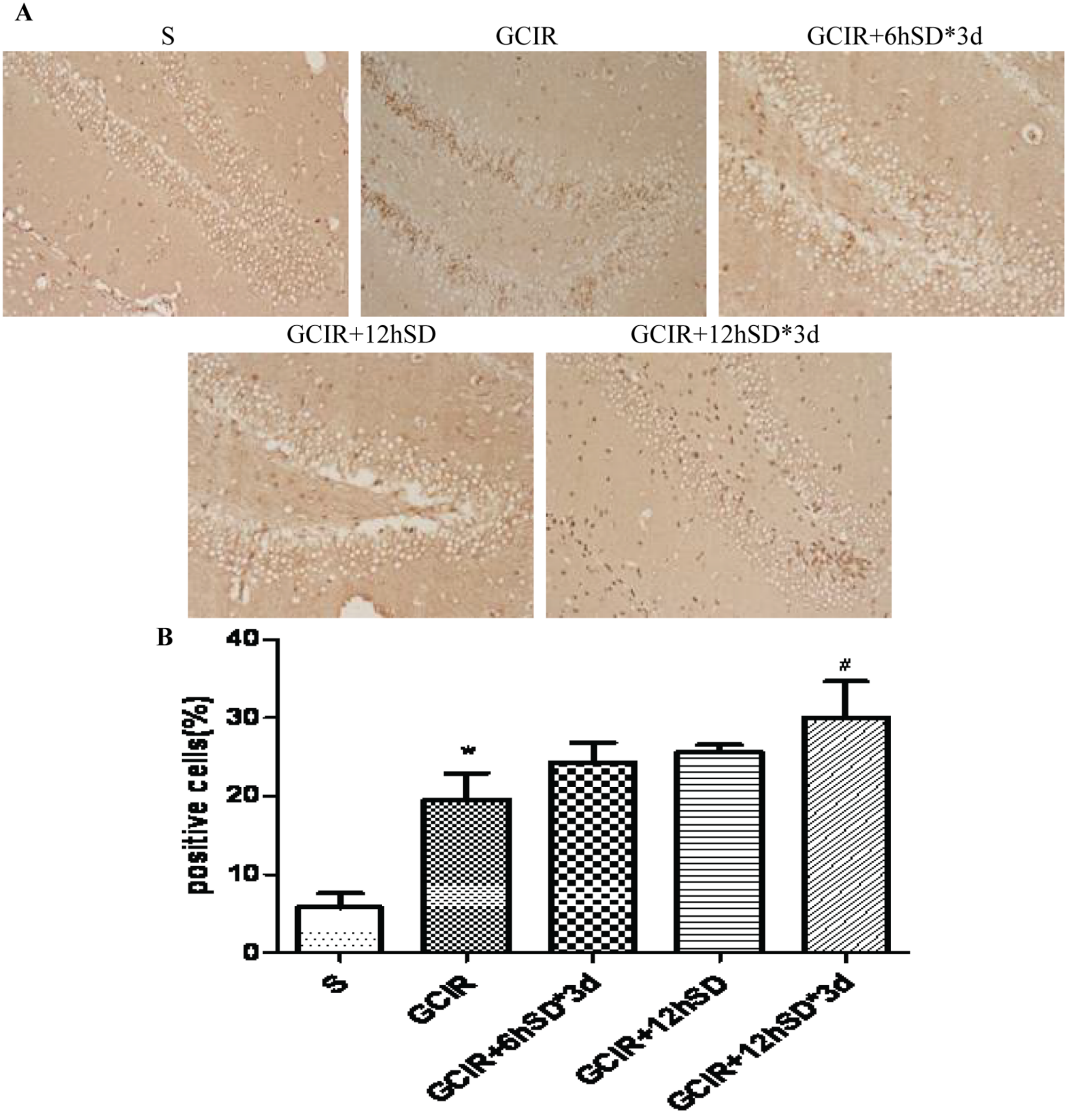
Effect of short-term SD on the BrdU-positive cells in the rat DG area 14 days after GCIR. Representative photomicrographs of BrdU-positive cells (brown) in the DG area shown at 200x magnification (A). Histogram representing the percentage of BrdU-positive cells in proportion of the total number of cells in the DG area of the rat hippocampus ($\bar{x}\pm \text{S}$, n = 4) (B). * P<0.01 compared with the S group; # P<0.05 compared with the GCIR group.

BrdU-positive cells were showed brown or black irregular granular nuclear markers distributed along the hippocampal dentate gyrus (DG) (Fig 3A). The results showed that a small number of BrdU-positive cells were observed in the hippocampal DG in the S group (Fig 3B). Relative to the S group, the number of BrdU-positive cells was substantially increased in the GCIR group (P<0.01) (Fig 3B). While the number of BrdU-positive cells had no significant difference in the GCIR+6hSD*3d group and GCIR+12h SD group compared with GCIR group (Fig 3B). Furthermore, the number of BrdU-positive cells in GCIR+12hSD*3d group was largely higher than that in the GCIR group(P<0.05) (Fig 3B).

### Effect of short-term sleep deprivation on cell differentiation in the hippocampal dentate gyrus in rats 28 d after GCIR

**Fig 4:**
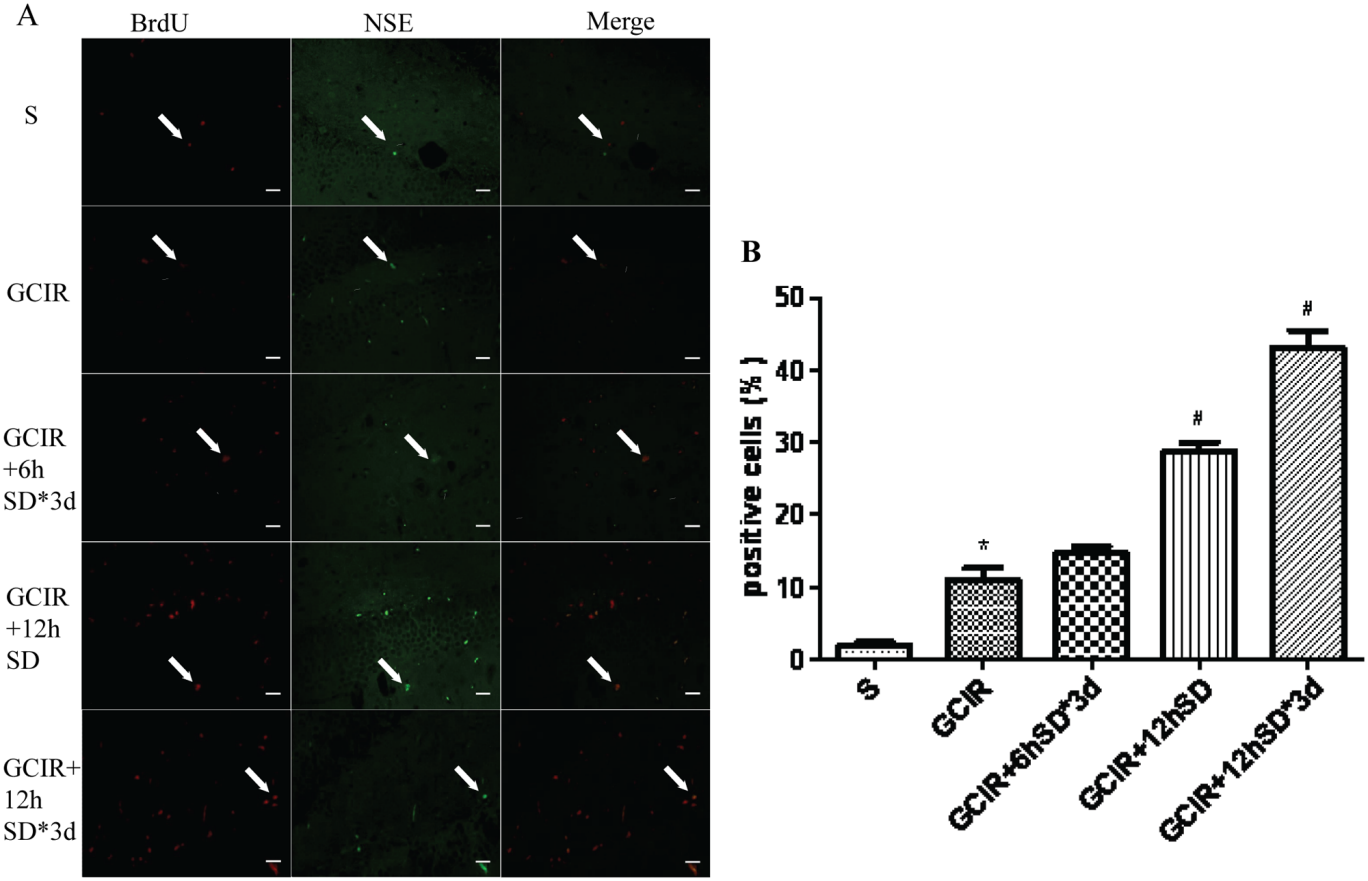
Effect of short-term SD on the BrdU/NSE-positive cells in the DG area of the rat hippocampus 28 days after GCIR. Representative photomicrographs of BrdU-positive (red), NSE-positive (green), and BrdU/NSE-positive cells (orange) in the hippocampal DG area shown at 400x magnification(A). Scale bar = 10μm. Histogram representing the percentage of BrdU/NSE-positive cells in total of BrdU-positive cells in the DG area of the rat hippocampus ($\bar{x}\pm \text{S}$, n = 4) (B). * P<0.01 compared with the S group; # P<0.05 compared with the GCIR group.

Four weeks after GCIR, BrdU/NSE double fluorescence-positive cells were rarely found in the S group (Fig 4A). However, the number of double-positive cells was significantly increased in the GCIR group compared to the S group (P<0.01) (Fig 4B). The BrdU/NSE double-positive number in the GCIR+6hSD*3d group had no difference compared to the GCIR group (Fig 4B). Moreover, a higher number of BrdU/NSE-positive cells was observed in short-term SD groups (including the GCIR+12hSD group and GCIR+12hSD*3d group) compared with the GCIR group (P<0.05) (Fig 4B).

### The expression of hippocampal BDNF in rats 7 d after GCIR

The expression of hippocampal BDNF in all short-term SD groups (including GCIR+6hSD*3d, GCIR+12hSD, and GCIR+12hSD*3d) was significantly increased relative to the GCIR group (P<0.05), and the most substantial increase was observed in the GCIR+12hSD*3d group (Table 2).

**Table 2:** BDNF changes in the hippocampus of the rat ($\bar{\text{x}}\pm \text{S}$, n = 6).

Groups	BDNF (pg/ml)
	Hippocampus
S	126.10±26.93
GCIR	153.70±76.05
GCIR+SD6h*3d	219.27±40.64^#^
GCIR+SD12h	252.99±50.95^#^
GCIR+SD12h*3d	290.67±42.69^#^

^#^ P<0.05 compared with the GCIR group.

## Discussion

In this study, we examined the beneficial effects of different durations of short-term SD on learning, memory function and neurogenesis in the hippocampus of rats exposed to GCIR. We found that after GCIR, (1) short-term SD could ameliorate the impairments in learning and memory, (2) short-term SD could promote the proliferation and differentiation of newly generated cells in the hippocampal DG, (3) the BDNF signaling pathway participated in the stimulation of neurogenesis, and (4) the GCIR+12hSD*3d-treated method may be the most appropriate way to implementation of sleep intervention in the rat with GCIR.

As we know the brain is highly sensitive to ischemia, and reperfusion exacerbates ischemic damage. The hippocampal neurons, particularly the pyramidal neurons in the CA1 region, are known to be the most sensitive to the deleterious effects of cerebral ischemia/reperfusion[28, 29]. And as the hippocampus is an important region for learning and memory in mammals[30], the majority of ischemia survivors suffer from various cognitive dysfunctions[31]. Using the current model, our study showed that global ischemia significantly compromised spatial learning and memory function.

In the present study, the rats in the GCIR+12hSD*3d group showed less escape latency in the five training days relative to the GCIR group, suggesting an improvement in spatial learning. And all groups of rats treated with short-term SD in our experiment spent significant longer and made substantial more frequent cross-platform movements in the original platform quadrant than the rats underwent GCIR alone in the probe test, suggesting an attenuation of memory dysfunction. In total, the group of rats subjected to 12 h SD for 3 d consistently showed the greatest recovery of cognitive function. Similar results have also been reported in the literature; for instance, Moldovan et al. found that rats with occlusion of the middle cerebral artery (MCAO) that were subjected to pretreatment with 6 h of SD showed better learning and memory performance during the first week of recovery; this was also consistent with reduced signs of morphological damage[13]. Additionally, Martinez-Vargas showed that 24 h of SD after a TBI had a neuroprotective effect by reducing morphological damage and enhancing recovery in rats[14]. The mechanism of how short-term SD promotes neurological recovery from brain injury is largely unclear. Some previous reports suggested that sleep deprivation prior to transient GCIR could attenuate hippocampal damage by attenuating inflammatory responses or glial scar formation [11, 12]. In the present study, we induced sleep deprivation intervention 48 h following GCIR, and focused on observing the neurogenesis effects of different durations of short-term SD.

A considerable degree of neurogenesis has been reported to occur following cerebral ischemia in the adult mammalian brain [32–34]. The regional correlation of neurogenesis in the hippocampal DG has also been studied in various disorders that cause memory impairment. Global ischemia stimulates cell proliferation in the hippocampal DG, which peaks at 1–2 weeks after ischemia [35, 36]. Many newly generated cells die in the weeks following ischemia, but the minority of cells that survive will differentiate into neurons approximately 4 weeks later[37]. In our study, compared with the GCIR group, a larger number of BrdU-positive cells was observed 14 days after GCIR in the hippocampal DG of the rats treated with short-term SD.NSE, a neuronal marker, can further determine the cell lineage of the newly generated cells. We also observed an increased number of BrdU/NSE-positive cells in the short-term SD groups, especially in the GCIR+12hSD group and GCIR+12hSD*3d group, 28 days after GCIR compared with the GCIR group. These results indicated that short-term SD not only promoted the proliferation of newly generated cells but also stimulated the proliferating cells to differentiate into neurons in the hippocampal DG. However, the most effective proliferation and differentiation occurred in the GCIR rats with 12 h SD for 3 successive days.

Our results are in agreement with previous studies indicating that sleep deprivation for relatively short periods(<48 h), such as 12 h, significantly stimulates neurogenesis in the hippocampus of normal rats by enhancing cell proliferation and the survival of newly generated cells[15, 16]. Based on these data, it may be tentatively concluded that delayed short-term SD (after 48 h) facilitates cell repair in the hippocampus through the stimulation of neurogenesis via endogenous progenitor cells to improve behavioral recovery and the most appropriate way is 12 h SD for 3 successive days.

In our experiment, the ELISA results showed that the BDNF level of hippocampal tissues was significantly increased after different periods of short-term SD 7 d following GCIR rats, which is in line with earlier reports suggesting that 12 h of short-term SD could increase the expression of hippocampal BDNF[38, 39]. Numerous evidences have also indicated that BDNF is required for basal neurogenesis in the hippocampal DG [40, 41]. The knockdown of BDNF by RNA interference and lentiviral-mediated gene silencing in the DG reduces hippocampal neurogenesis[42]. Therefore, BDNF signaling is one possible mechanism for short-term SD-induced neurogenesis, and further research is needed to clarify the down-stream signaling mechanisms.

## Conclusion

The current study clearly demonstrates that 48 h delayed short-term SD, especially the GCIR+12hSD*3d-treated method, is effective in promoting neurogenesis in the hippocampal DG and facilitating cognitive recovery following GCIR in the adult rat. Additionally, the underlying mechanism of short-term SD in neurogenesis may be mediated by the BDNF signaling pathway. These results suggest that short-term SD in an appropriate way may be a potential strategy for the clinical treatment of ischemic injury in brain tissues.
